# Association between tuberculosis and psychotic experiences: Mediating factors and implications for patient care in low- and middle-income countries

**DOI:** 10.7189/jogh.14.04005

**Published:** 2024-03-01

**Authors:** Anna Monistrol-Mula, Mireia Felez-Nobrega, Hans Oh, Josep Maria Haro, Ai Koyanagi

**Affiliations:** 1 Group of Epidemiology of Mental Disorders and Ageing, Sant Joan de Déu Research Institute, Esplugues de Llobregat, (Barcelona) Spain; 2 Research, Teaching, and Innovation Unit, Parc Sanitari Sant Joan de Déu, Sant Boi de Llobregat, Barcelona, Spain; 3 Centro de Investigación Biomédica en Red de Salud Mental (CIBERSAM), Instituto de Salud Carlos III, Madrid, Spain; 4 Department of Medicine, Universitat de Barcelona, Barcelona, Spain; 5 Suzanne Dworak Peck School of Social Work, University of Southern California, Los Angeles, California, USA

## Abstract

**Background:**

Tuberculosis may play a role in the aetiology of psychosis. However, little is known about the association between tuberculosis and psychotic experiences (PEs) or the mediating factors of this association.

**Methods:**

We analysed cross-sectional data from 48 low- and middle-income countries of the World Health Survey (WHS). Tuberculosis assessment was based on past 12-month symptoms of active tuberculosis. We assessed four types of past 12-month PEs with the Composite International Diagnostic Interview. We performed multivariable multinomial logistic regression and mediation analysis.

**Results:**

We analysed data on 224 842 individuals aged ≥18 years (mean age = 38.3 years, standard deviation = 16.0; 50.7% women). Tuberculosis was associated with 1.84 (95% confidence interval (CI) = 1.41–2.40), 2.18 (95%CI = 1.58–3.03), and 3.79 (95%CI = 2.88–4.98) times higher odds for 1, 2, and ≥3 PEs, respectively. The mediation analysis showed that the association between tuberculosis and at least one PE is mainly explained by anxiety (31.5%), sleep/energy (27.8%), and pain/discomfort (23.5%).

**Conclusions:**

Tuberculosis was associated with increased odds of PEs. Factors such as affect, sleep, and pain should be considered in tuberculosis patients to target those who might be particularly vulnerable to PEs, and consequently, to psychotic disorders and other adverse effects of PE.

Psychotic experiences (PEs) are psychotic symptoms (i.e. hallucinations and delusions) that occur at a sub-clinical level. They commonly occur in the general population (approximately 5–7% of adults report lifetime PEs) [1]. Although in most cases these experiences are transient, up to 7% of individuals with PE will eventually develop a psychotic disorder [2]. Currently, there is growing evidence reporting that PEs in adulthood are associated with a myriad of adverse health outcomes, including higher odds for non-psychotic mental disorders [3], physical health conditions [4], and premature mortality [5].

Current literature suggests that infections could play a role in the aetiology of some mental disorders, particularly psychotic disorders [6]. One study examined whether infectious were related to lifetime PEs; while its findings suggested infections were associated with greater odds of PEs, the associations were not statistically significant [7]. Another study found that coronavirus disease 2019 (COVID-19) infection was associated with greater odds of PEs [8]. To date, there are limited data on the association between PE and infectious diseases such as tuberculosis, since previous studies on PEs and physical diseases have predominantly focussed on non-communicable diseases only.

Tuberculosis is a contagious bacterial disease that figures as the second-deadliest infectious illness globally, surpassed only by COVID-19. It constitutes an especially large burden in low- and middle-income countries (LMICs), as 98% of tuberculosis cases and 80% of tuberculosis deaths occur in these regions [9]. It has been hypothesised that tuberculosis increases the risk for PE via biological mechanisms such as inflammation, which can lead to impairments in brain function including the dysregulation of dopamine pathways [10]. In fact, studies have found an association between tuberculosis and a higher risk of developing a psychotic disorder [11,12]. For instance, one national cohort study with more than 14 000 participants found that tuberculosis infection in childhood was associated with increased risk for both schizophrenia and affective psychosis [11]. Alternatively, tuberculosis can increase risk for PEs via factors such as disability, insomnia, psychological distress, and pain, which can all be the consequence of tuberculosis, and also risk factors for PE [12]. However, to date, no studies have focussed specifically on the association between tuberculosis and PEs, while the hypothesised mechanisms underlying this association are predominantly theory-based. Given that PEs are part of a continuum of psychosis [13], studying early subclinical manifestations of psychosis related to tuberculosis might also shed light on the aetiology of psychosis.

Considering the well-established connection between infections and psychosis, we expected to observe an association between tuberculosis infection and increased odds of PEs. Thus, we sought to examine the relationship between tuberculosis and PE and quantify the extent to which this association could be explained by factors such as affect, cognition, interpersonal activities, mobility, pain/discomfort, perceived stress, self-care, and sleep/energy. Exploring this association is particularly relevant in the context of LMICs, where tuberculosis is still a major cause of morbidity and mortality [14], and where the prevalence of PE has been reported to be high [15].

## METHODS

### The survey

This is a cross-sectional study that analysed data from the World Health Survey (WHS), which was conducted in 70 countries between 2002–04, as detailed elsewhere [16]. Briefly, the WHS conducted single-stage random sampling 10 and multi-stage random cluster sampling in 60 countries. For each household, one adult respondent was randomly selected utilising Kish tables. The questionnaire was translated and back-translated following standard procedures to ensure comparability between countries, and face-to-face interviews were conducted. The overall individual response rate was 98.5%. To adjust for non-response, sampling weights were created using the population distribution based on data of the United Nations Statistical Division. Ethical approval for the survey was given by ethical boards at each study site, and all participants gave their informed consent.

Out of the 70 countries, 69 had publicly available data. Of these, we excluded 10 countries from the analysis due to a lack of sampling information. Furthermore, we omitted 10 high-income countries as most of them used a shorter version of the questionnaire which did not collect information on PE, and also because our study focussed on LMICs. We also excluded Turkey specifically due to a lack of data on PE. Thus, the final sample consisted of 48 countries, which corresponded to 21 low-income and 27 middle-income countries, according to the World Bank classification at the time of the survey (2003) [17]. Based on the United Nations’ classification system [18], these corresponded to 20 countries in Africa, 6 in the Americas, 13 in Asia, and 9 in Europe. The data were nationally representative for all but six countries (China, Comoros, the Republic of Congo, Ivory Coast, India, and Russia) (Table S1 in the **Online Supplementary Document**).

### Variables

#### Tuberculosis

Sputum smear examinations or mycobacterial culture were not performed in the WHS; consequently, as was the case with previous published studies which used the same data set, we based the assessment of tuberculosis on past 12-month symptoms of active tuberculosis [19]. Specifically, we considered individuals with a cough lasting for three weeks or more and who were coughing up blood (or had blood in phlegm) to have active tuberculosis. Prior studies have shown that the presence of these symptoms has a sensitivity of 65–93% and a specificity of 55–87% to detect tuberculosis [19-21].

#### Psychotic experiences

Positive psychotic symptoms were self-reported using the module of the WHO Composite International Diagnostic Interview (CIDI) 3.0 [22], which is highly consistent with clinician ratings [23]. Participants were asked the following yes/no questions:

‘During the last 12 months, have you experienced’:

− ‘A feeling that your thoughts were being directly interfered or controlled by another person, or your mind was being taken over by strange forces?’ (delusions of control);
− ‘An experience of seeing visions or hearing voices that others could not see or hear when you were not half asleep, dreaming or under the influence of alcohol or drugs?’ (hallucinations);
− ‘A feeling something strange and unexplainable was going on that other people would find hard to believe?’ (delusional mood);
− ‘A feeling that people were too interested in you or there was a plot to harm you?’ (delusions of reference and persecution).

The ‘hallucinations’ question did not refer to sleep-related states or substance use-associated conditions. Individuals who answered ‘yes’ to at least one of the above-mentioned questions were considered to have any PE. The number of PEs was summed and categorised as 0, 1, 2, and ≥3.

We excluded from all analyses those individuals with a self-reported lifetime diagnosis of psychosis (n = 2424), as PE by meaning does not include conditions that reach the diagnostic threshold.

### Confounding variables

Confounding variables included age, sex, wealth, education, setting (urban or rural), self-reported lifetime diabetes diagnosis, alcohol consumption, and smoking. We performed principal component analysis based on 15-20 assets to create country-wise wealth quintiles. Men who reported consuming ≥5 drinks and women consuming ≥4 drinks on one or two days in the past seven days were considered infrequent heavy drinkers, while people who drank these quantities in ≥3 days, in the past week were considered frequent heavy drinkers. All other respondents (excluding lifetime abstainers) were considered non-heavy drinkers. Current smoking was dichotomised as ‘yes’ and ‘No.’

### Mediators

We based our selection of potential mediators in the association between tuberculosis and PE on past literature and included affect, cognition, interpersonal activities, mobility, pain/discomfort, perceived stress, self-care, and sleep/energy. We utilised two questions each to measure the degree or severity of these potential mediators in the past 30 days (Table S2 in the **Online Supplementary Document**), scoring them individually on a five-point scale ranging from ‘none’ to ‘extreme/cannot do’ or ‘never’ to ‘very often’ (perceived stress). Using these two questions for each condition, we conducted factor analysis with polychoric correlations to estimate a factor score that was converted to scores ranging from 0 to 100, with greater values representing worse status [24]. Data on perceived stress were missing in Zimbabwe, Hungary and Brazil.

### Statistical analysis

We conducted the statistical analyses in Stata, version 14.2 (Stata Corp LP, College station, Texas). We tested the differences in sample characteristics by χ^2^ tests for categorical and Student’s *t*-tests for continuous variables. We also performed multivariable multinomial logistic regression analyses to assess the association between tuberculosis (exposure) and different types of PE (outcome). Furthermore, we estimated the association between tuberculosis and any PE by binary logistic regression using the overall sample and samples stratified by country income level (i.e. low-income, middle-income), and region (Africa, the Americas, Asia, Europe). We similarly assessed the association between tuberculosis and each type of PE. Finally, to determine the extent to which various factors may explain the relation between tuberculosis and any PE, we performed a mediation analysis using the ‘khb’ (Karlson Holm Breen) command in Stata [25]. This method decomposes the full effect of a variable into direct (the effect of tuberculosis on PE adjusted for the mediator) and indirect effects (the mediational effect). Using this method, we were able to calculate which percentage of the main association is explained by the mediator. We put each potential mediator in the model individually, and then in a model where all potential mediators were included simultaneously.

We adjusted all regression analyses for age, sex, wealth, education, country, setting, diabetes, alcohol consumption, and smoking, and took into account the sample weighting and the complex study design in all analyses. We presented the results from the regression analyses as odds ratios (ORs) with 95% confidence intervals (CIs). The level of statistical significance was set at *P* < 0.05.

## RESULTS

The analytical sample consisted of 224 842 individuals (50.7% women) aged ≥18 years, with a mean age of 38.3 years (standard deviation (SD) = 16.0) (**Table 1**). The prevalence of any PE (i.e. at least one type of PE) was 13.8%, while the prevalence of tuberculosis was 1.6%. The prevalence of any PE was much higher among those with (30.7%) compared to those without tuberculosis (12.8%). Specifically, the prevalence of ≥3 types of PEs was much higher among those with tuberculosis (11.6%) than in those without (3.0%). These results were corroborated in the multivariable multinomial logistic regression analysis, where tuberculosis was associated with 1.84 (95% CI = 1.41–2.40), 2.18 (95% CI = 1.58–3.03), and 3.79 (95% CI = 2.88–4.98) times higher odds for 1, 2, and ≥3 PEs, respectively (**Table 2**). Furthermore, based on the multivariable binary logistic regression, tuberculosis was associated with 2.31 (95% CI = 1.94–2.76) times higher odds for any PE in the overall sample. This association was significant in both low-income and middle-income countries, and all continents but Europe (**Table 3**). Tuberculosis was significantly associated with all individual types of PE: hallucinations (OR = 2.61; 95% CI = 2.05–3.31); delusions of reference and persecution (OR = 2.11; 95% CI = 1.68–2.64), delusional mood (OR = 2.37; 95% CI = 1.92–2.92), and delusions of control (OR = 2.63; 95% CI = 2.05–3.38) (data only shown here). Finally, the mediation analysis showed that affect mediated the largest proportion of the association between tuberculosis and any PE (31.5%), followed by sleep/energy (27.8%), and pain/discomfort (23.5%), while the other factors explained less than 20% of the association (cognition: 17.9%, mobility: 14.6%, perceived stress: 12.0%, interpersonal activities: 8.3%, self-care: 7.4%). These potential mediators collectively explained 50.8% of the association (**Table 4**).

**Table 1:** Sample characteristics (overall and by tuberculosis status)*

	Tuberculosis			
**Characteristic**	**Overall**	**No**	**Yes**	***P*-value†**
**Age in years, x̄ (SD)**	38.3 (16.0)	38.4 (16.0)	43.3 (17.1)	<0.001
**Sex**				0.005
Men	49.3	49.1	54.7	
Women	50.7	50.9	45.3	
**Wealth**				<0.001
Poorest	20.0	20.1	31.3	
Poorer	19.9	19.9	22.4	
Middle	19.9	20.0	18.0	
Richer	20.1	20.0	16.4	
Richest	20.2	20.1	11.9	
**Education**				<0.001
No formal	25.8	24.3	43.5	
Primary	30.9	31.8	34.2	
Secondary	34.0	33.7	19.9	
Tertiary	9.3	10.1	2.3	
**Diabetes**				<0.001
No	97.0	97.0	93.5	
Yes	3.0	3.0	6.5	
**Setting**				<0.001
Rural	56.0	54.8	65.7	
Urban	44.0	45.2	34.3	
**Current smoking**				<0.001
No	73.5	73.0	62.7	
Yes	26.5	27.0	37.3	
**Alcohol consumption**				0.032
Lifetime abstainer	66.1	66.2	65.3	
Non-heavy	29.1	28.9	29.4	
Infrequent heavy	3.8	3.9	3.3	
Frequent heavy	1.0	1.1	2.0	

SD – standard deviation, x̄ – mean

*Data presented as percentages unless stated otherwise.

†Calculated by χ^2^ tests, except for age (Student’s *t* test).

**Table 2:** Association between tuberculosis and the number of different types of psychotic experiences estimated by multivariable multinomial logistic regression*

	Total number of different types of psychotic experiences (outcome)					
	**1 vs 0**		**2 vs 0**		**≥3 vs 0**	
**Characteristic**	**OR (95% CI)**	***P*-value**	**OR (95% CI)**	***P*-value**	**OR (95% CI)**	***P*-value**
**Tuberculosis – yes vs no**	1.84 (1.41–1.00)	<0.001	2.18 (1.58–3.03)	<0.001	3.79 (2.88–4.98*	<0.001
**Age in years, per unit increase**	1.00 (0.99–1.00)	<0.05	1.00 (0.99–1.00)	NS	1.00 (1.00–1.00)	NS
**Sex – women vs Men**	1.33 (1.21–1.46)	<0.001	1.36 (1.19–1.56)	<0.001	1.54 (1.23–1.92)	<0.001
**Wealth**						
Poorest	1.00		1.00		1.00	
Poorer	1.16 (1.01–1.33)	<0.05	1.09 (0.94–1.27)	NS	0.84 (0.71–0.98)	<0.05
Middle	0.99 (0.87–1.13)	NS	1.03 (0.87–1.22)	NS	0.82 (0.69–0.96)	<0.05
Richer	1.01 (0.87–1.17)	NS	0.89 (0.74–1.07)	NS	0.82 (0.59–1.13)	NS
Richest	0.93 (0.79–1.10)	NS	0.95 (0.78–1.16)	NS	0.55 (0.40–0.075)	<0.001
**Education**						
No formal	1.00		1.00		1.00	
Primary	1.29 (1.12–1.47)	<0.001	1.12 (0.95–1.32)	NS	1.15 (0.96–1.37)	NS
Secondary	1.27 (1.06–1.51)	<0.01	1.01 (0.82–1.25)	NS	0.78 (0.62–0.99)	<0.05
Tertiary	1.00 (0.80–1.26)	NS	0.85 (0.63–1.17)	NS	1.11 (0.49–2.49)	NS
**Diabetes – yes vs no**	1.66 (1.37–2.02)	<0.001	1.37 (1.03–1.82)	<0.05	2.32 (1.82–2.95)	<0.001
**Setting – urban vs rural**	1.04 (0.91–1.19)	NS	1.07 (0.92–1.26)	NS	1.04 (0.87–1.25)	NS
Current smoking – yes vs no	1.19 (1.07–1.31)	<0.01	1.24 (1.07–1.43)	<0.01	1.37 (1.13–1.66)	<0.01
**Alcohol consumption**						
Lifetime abstainer	1.00		1.00		1.00	
Non-heavy	1.30 (1.15–1.48)	<0.001	1.39 (1.20–1.62)	<0.001	1.26 (1.04–1.52)	<0.05
Infrequent heavy	1.33 (1.07–1-66)	<0.05	1.83 (1.40–2.40)	<0.001	1.92 (1.41–2.63)	<0.001
Frequent heavy	1.25 (0.85–1.82)	NS	1.71 (1.07–2.76)	<0.05	1.61 (0.98–2.64)	NS

CI – confidence interval, NS – not significant, OR – odds ratio

*Model is mutually adjusted for all variables in the table and country.

**Table 3:** Association between tuberculosis (exposure) and any psychotic experience (outcome) estimated by multivariable binary logistic regression (overall, and by regions or country income level)*

Sample	OR (95% CI)	*P*-value
Overall	2.31 (1.94–2.76)	<0.001
Low-income countries	1.89 (1.46–2.45)	<0.001
Middle-income countries	2.95 (2.32–3.75)	<0.001
Africa	2.33 (1.79–3.03)	<0.001
The Americas	3.06 (2.16–4.35)	<0.001
Asia	1.95 (1.42–2.67)	<0.001
Europe	1.19 (0.35–4.12)	NS

CI – confidence interval, NS – not significant, OR – odds ratio

*Any psychotic experience referred to having at least one delusional mood, delusions of reference, and persecution, delusions of control, and hallucinations in the past 12 mo. Models are adjusted for age, sex, wealth, education, setting, diabetes, smoking, alcohol consumption, and country.

**Table 4:** Mediators in the association between tuberculosis and any psychotic experience*

Mediator	Total effect, OR (95%CI)	*P*-value	Direct effect, OR (95%CI)	*P*-value	Indirect effect, OR (95%CI)	*P*-value	Mediated %
Affect	2.29 (1.89–2.77)	<0.001	1.76 (1.45–2.13)	<0.001	1.30 (1.22–1.38)	<0.001	31.5
Cognition	2.32 (1.94–2.77)	<0.001	1.99 (1.66–2.39)	<0.001	1.16 (1.12–1.21)	<0.001	17.9
Interpersonal activities	2.32 (1.94–2.78)	<0.001	2.17 (1.81–2.59)	<0.001	1.07 (1.04–1.10)	<0.001	8.3
Mobility	2.34 (1.95–2.80)	<0.001	2.06 (1.72–2.48)	<0.001	1.13 (1.09–1.17)	<0.001	14.6
Pain/discomfort	2.35 (1.96–2.81)	<0.001	1.92 (1.60–2.31)	<0.001	1.22 (1.17–1.27)	<0.001	23.5
Perceived stress†	2.12 (1.74–2.58)	<0.001	1.93 (1.59–2.36)	<0.001	1.09 (1.05–1.14)	<0.001	12.0
Self-care	2.32 (1.94–2.77)	<0.001	2.18 (1.82–2.61)	<0.001	1.06 (1.04–1.09)	<0.001	7.4
Sleep/energy	2.32 (1.93–2.78)	<0.001	1.83 (1.53–2.20)	<0.001	1.26 (1.20–1.32)	<0.001	27.8
All mediators†	1.97 (1.60–2.42)	<0.001	1.39 (1.13–1.71)	0.002	1.41 (1.31–1.52)	<0.001	50.8

CI – confidence interval, OR – odds ratio

*Any psychotic experience referred to having at least one delusional mood, delusions of reference, and persecution, delusions of control, and hallucinations in the past 12 mo. Models are adjusted for age, sex, wealth, education, setting, diabetes, smoking, alcohol consumption, and country.

†Data were not available from Brazil, Hungary, and Zimbabwe.

## DISCUSSION

### Main findings

In our large nationally representative sample of adults from 48 LMICs, we found that tuberculosis was related to more than two-fold increased odds for at least one PE; this association was particularly pronounced for ≥3 PEs (OR = 3.79). This finding remained mostly consistent by income levels and across continents, although the association in Europe was not significant. The mediation analysis identified affect, sleep/energy, and pain/discomfort as potentially important factors in this association, while cognition, mobility, perceived stress, interpersonal activities, and self-care played a less important role. Collectively, all these potential mediators explained about half of the association between tuberculosis and at least one PE. To the best of our knowledge, this is the first study specifically on the association between tuberculosis and PE, and the first to quantify the extent to which a variety of factors may explain this association.

### Interpretation of findings

Our findings on the association between tuberculosis and PEs are in line with a cross-sectional study performed in Nigeria reporting an increased prevalence of both schizophrenia and affective psychosis in patients with tuberculosis [12], and with a British longitudinal study reporting an increased risk of psychotic disorders in adulthood following a tuberculosis infection in childhood [11]. Moreover, our results support findings from several meta-analyses reporting increased rates of tuberculosis among patients with psychotic disorders and subclinical psychosis [26]. Although data on psychotic disorders cannot be directly extrapolated to PEs, a clinical continuum between PEs and psychotic disorders has been demonstrated, while risk factors for PEs, including genetic factors, overlap with those for schizophrenia and other psychosis [27]. In fact, our data showed that tuberculosis was associated with particularly increased odds for ≥3 PEs, and the persistence and severity of symptoms (greater number of PEs could be a proxy of this) has been reported to be one key factor for the transition to clinical psychosis [13].

Our study showed that affect explained the largest proportion of the tuberculosis-PE association, followed by sleep/energy, and pain/discomfort. Tuberculosis could increase the risk for onset of anxiety and depressive symptoms through biological mechanisms such as increases in systemic inflammation [28], hypothalamic-pituitary-adrenal (HPA) axis dysregulation [29], or the influence of anti-tuberculosis drugs [30], while the same neurobiological causes of affective disorders could also lead to PEs [31]. Alternatively, affective disorders could be the consequence of some symptoms of tuberculosis (e.g. chronic cough, hypoxia, fatigue) [32] or social stressors linked to the disease such as stigma, social isolation, or poverty [33]. In turn, PEs might result from the cognitive processes and emotions associated with affective disorders. For instance, anxiety processes such as meta-worry could be important for the formation of persecutory delusions, while depression and low self-esteem could lead to reference delusions [34,35].

In terms of sleep problems, current literature suggests that most tuberculosis patients have poor sleep quality [36]. Tuberculosis may impair sleep through the immune-inflammatory response to the infection [37], while the perceived stress resulting from stigma or economic problems related to tuberculosis could activate the release of cortisol by the HPA axis, leading to sleep dysfunctions [38,39]. Sleep disturbance is strongly linked to PEs [40], possibly through several mechanisms including neuroplasticity changes [41] and dopamine/glutamate alterations [42]. Furthermore, pain can result from common tuberculosis symptoms such as cough [43], as well as from pulmonary and extrapulmonary complications of the disease [44]. In turn, perceived stress, disability (e.g. mobility problems), and sleep problems arising from pain [40,45,46] might set a favourable environment for the onset of PEs.

These factors, together with all other significant factors found in this study (cognition, mobility, perceived stress, interpersonal activities, and self-care), only explained 50.8% of the association between tuberculosis and PEs. This association could possibly be further explained by other important factors such as systemic inflammation triggered by the bacterial infection [47] or anti-tuberculosis medication [30]. Future studies may seek to identify additional factors.

Finally, the association between tuberculosis and PEs was significant across all continents but Europe. This lack of association is probably due to the lower incidence of tuberculosis in Europe compared to other continents [48], together with a lack of statistical power, as our sample size for this subpopulation was approximately four times smaller than that for the other continents. However, other factors might also have contributed to these results, such as differences in the use/availability of anti-tuberculosis drugs; a better monitoring of their psychiatric adverse events; or a wider availability of services for mental health problems in Europe, together with better treatment options for other important factors identified in this study, such as sleep problems or pain.

### Clinical implications

Although further longitudinal studies are required, our findings suggest that addressing the co-existing conditions of tuberculosis (e.g. affective symptoms, sleep problems, pain) might contribute to preventing the onset of PEs and its related adverse outcomes in patients with tuberculosis. Results from recent studies suggest that psychological interventions might be effective in addressing affective and sleep problems, and subsequently preventing PEs in tuberculosis patients. One randomised controlled trial (RCT) reported that two months of cognitive-behavioural therapy (CBT) improved quality of life, anxiety, and depressive symptoms in patients with tuberculosis [49]. Although there is no literature on the effectiveness of psychological interventions in preventing PEs in tuberculosis patients, several RCTs have reported a reduction of PEs following CBT in people at high risk of psychosis [50]. Additionally, RCTs focused on improving sleep problems through CBT have reported a reduction of PEs after the therapy [51]. Besides, psychological interventions could have further benefits in patients with tuberculosis, such as improving treatment adherence and clinical outcomes [52]. Therefore, the inclusion of psychological interventions in tuberculosis treatment programmes could potentially improve not only PEs and other mental health problems related to tuberculosis, but also treatment and disease outcomes. Moreover, given the common comorbidity of tuberculosis and mental disorders, the improvement of general mental health care services might help to reduce the global incidence of tuberculosis [26], while public health efforts to tackle tuberculosis could have a beneficial impact on global mental health [30].

### Strengths and limitations

The major strength of our study is the use of a large, predominantly nationally representative data set from different LMICs across several continents. Nevertheless, our results should be interpreted in light of potential limitations. First, as data were self-reported, reporting bias may exist. Second, tuberculosis was not based on a laboratory-confirmed diagnosis, but rather on the presence of two symptoms of active tuberculosis during the past 12 months, which have shown to account for a sensitivity and specificity of 65–93% and 55–87%, respectively, for detecting tuberculosis. This might have led to misclassification in some cases. Third, we overlooked potential PEs happening after mycobacterium tuberculosis has been eradicated from the body. Fourth, we lacked information regarding the use of anti-tuberculosis drugs and were therefore unable to assess their influence on the association between tuberculosis and PEs. Fifth, data used in this study were collected in 2003, and LMICs were categorised following the World Bank classification at that time. Thus, it is probable that the epidemiology and demographics of many included countries have changed, which means our results might not reflect the current circumstances. Finally, due to the cross-sectional design, the directionality of the reported association cannot be deduced readily. Relatedly, we might have overestimated the mediated percentage in our study, given the various ways in which the potential mediators assessed here can be linked with both tuberculosis and PEs.

## CONCLUSION

Tuberculosis was related to higher odds for PEs among adults aged ≥18 years in LMICs, while affect, sleep problems, and pain were the main mediators of this association. Future studies are warranted to understand the underlying mechanisms of this association; in particular, longitudinal studies are necessary to understand temporal associations. Affect, sleep, and pain should be considered in tuberculosis patients in order to target individuals at high risk of developing PE, who are thus vulnerable to psychotic disorders and other adverse effects of PE.

## Additional material


Online Supplementary Document
