# Supplementary material for: Association between tuberculosis and psychotic experiences: Mediating factors and implications for patient care in low- and middle-income countries
Source: J Glob Health. 2024 Mar 1;14:04005. doi: 10.7189/jogh.14.04005 (PMC10902804; doi:10.7189/jogh.14.04005)
Supplement: Online Supplementary Document [file jogh-14-04005-s001.pdf]

Supplementary Material

Table S1. Country-income level, region, and sample size of each country

Country	Country-income level	Region	N
Bangladesh	Low-income	Asia	5,423
Bosnia & Herzegovina	Middle-income	Europe	1,014
Brazil	Middle-income	the Americas	4,889
Burkina Faso	Low-income	Africa	4,755
Chad	Low-income	Africa	4,222
China	Middle-income	Asia	3,953
Comoros	Low-income	Africa	1,730
Croatia	Middle-income	Europe	958
Czech Republic	Middle-income	Europe	910
Dominican Republic	Middle-income	the Americas	4,436
Ecuador	Middle-income	the Americas	4,395
Estonia	Middle-income	Europe	994
Ethiopia	Low-income	Africa	4,605
Georgia	Middle-income	Asia	2,720
Ghana	Low-income	Africa	3,880
Hungary	Middle-income	Europe	1,382

India	Low-income	Asia	9,222
Ivory Coast	Low-income	Africa	3,030
Kazakhstan	Middle-income	Asia	4,468
Kenya	Low-income	Africa	4,324
Laos	Low-income	Asia	4,690
Latvia	Middle-income	Europe	845
Malawi	Low-income	Africa	5,203
Malaysia	Middle-income	Asia	5,963
Mali	Low-income	Africa	3,535
Mauritania	Low-income	Africa	3,535
Mauritius	Middle-income	Africa	3,848
Mexico	Middle-income	the Americas	38,559
Morocco	Middle-income	Africa	4,964
Myanmar	Low-income	Asia	5,851
Namibia	Middle-income	Africa	3,852
Nepal	Low-income	Asia	8,383
Pakistan	Low-income	Asia	5,945
Paraguay	Middle-income	the Americas	5,088
Philippines	Middle-income	Asia	9,990
Republic of Congo	Low-income	Africa	2,015
Russia	Middle-income	Europe	4,304

Senegal	Low-income	Africa	2,840
Slovakia	Middle-income	Europe	1,802
South Africa	Middle-income	Africa	2,253
Sri Lanka	Middle-income	Asia	6,394
Swaziland	Middle-income	Africa	1,917
Tunisia	Middle-income	Africa	4,903
Ukraine	Middle-income	Europe	2,793
Uruguay	Middle-income	the Americas	2,947
Vietnam	Low-income	Asia	3,429
Zambia	Low-income	Africa	3,767
Zimbabwe	Low-income	Africa	3,917

Table S2. Questions used to assess health status and perceived stress

**Mobility**	(1) Overall in the last 30 days, how much difficulty did you have with moving around?<br><br>(2) In the last 30 days, how much difficulty did you have in vigorous activities, such as running 3 km (or equivalent) or cycling?
**Self-care**	(1) Overall in the last 30 days, how much difficulty did you have with self- care, such as washing or dressing yourself?<br><br>(2) In the last 30 days, how much difficulty did you have in taking care of and maintaining your general appearance (e.g. grooming, looking neat and tidy etc.)
**Pain and discomfort**	(1) Overall in the last 30 days, how much of bodily aches or pains did you have?<br><br>(2) In the last 30 days, how much bodily discomfort did you have?
**Cognition**	(1) Overall in the last 30 days, how much difficulty did you have with concentrating or remembering things?<br><br>(2) In the last 30 days, how much difficulty did you have in learning a new task (for example, learning how to get to a new place, learning a new game, learning a new recipe etc.)?
**Interpersonal activities**	(1) Overall in the last 30 days, how much difficulty did you have with personal relationship or participation in the community?<br><br>(2) In the last 30 days, how much difficulty did you have in dealing with conflicts and tensions with others?
**Sleep and energy**	(1) Overall in the last 30 days, how much of a problem did you have with sleeping, such as falling asleep, waking up frequently during the night or waking up too early in the morning?<br><br>(2) In the last 30 days, how much of a problem did you have due to not feeling rested and refreshed during the day (e.g. feeling tired, not having energy)?
**Affect**	(1) Overall in the last 30 days, how much of a problem did you have with feeling sad, low or depressed?<br><br>(2) Overall in the last 30 days, how much of a problem did you have with worry or anxiety?
**Perceived stress**	(1) In the last month, how often have you felt that you were unable to control the important things in your life?

	(2) In the last month, how often have you found that you could not cope with all the things that you had to do?
